# Human Papillomaviruses and Papillomatosis Lesions of the Female Lower Genital Tract

**DOI:** 10.1155/S1064744994000153

**Published:** 1994

**Authors:** Yu-Liang Fu, Yao-Xiong Hu, Han-Liang Ling, Zhen-Zhong Ye, Tian Liang, Mei-Gui Zhang, Yun-Ke Liu, Biao Kang, Yuan-Ji Luo, Shu-Ying He, Yong-Jian Lian

**Affiliations:** 1 Guangzhou Maternal and Neonatal Hospital Guangzhou China; 2 Guangzhou Medicinal Institute Guangzhou China; 3 Sun Yat Sen University of Medical Sciences Guangzhou China; 4 Guangzhou Medical College Guangzhou China; 5 Guangzhou Maternal and Neonatal Hospital 402 Renminzong Road Guangzhou 510180 China

## Abstract

*Objective:* The objective of this study was to determine whether human papillomavirus (HPV) infections are involved in the development of papillomatosis lesions of the lower female genital tract.

*Methods:* A total of 616 biopsy specimens of genital papillomatous lesions (307 nodular and 309 papular types) from 598 patients were anaylyzed for the presence of HPV DNA sequences by polymerase chain reaction (PCR). These specimens were also examined by histopathological assessment for characteristic HPV-associated cytological changes, by immunohistochemical staining for HPV-associated antigen, and by electron microscopy for the presence of virions.

*Results:* HPV DNA sequences were found in 97.9% (140 of 143 cases) and 1.1% (1 of 91 cases) of the nodular and papular papillomatosis cases tested, respectively. In 18 patients who had both types of papillomatosis lesions, HPV DNA was invariably found only in nodular tissues. HPV-associated antigen, koilocytosis, and virions were found in 53.6% (98 of 183 cases), 70.5% (129 of 183 cases), and 5.9% (5 of 85 cases) of nodular papillomatosis lesions tested, respectively.

*Conclusions:* These data suggest that nodular papillomatosis was closely associated with HPV infection, but that papular papillomatosis of the lower female genital tract may have an etiology other than HPV infection,


Infectious Diseases in Obstetrics and Gynecology 1:235–241 (1994)
© 1994 Wiley-Liss, Inc.

### Human Papillomaviruses and Papillomatosis Lesions of the Female Lower Genital Tract

**Yu-Liang Fu, Yao-Xiong Hu, Han-Liang Ling, Zhen-Zhong Ye, Tian Liang, Mei-Gui Zhang, Yun-Ke Liu, Biao Kang, Yuan-Ji Luo, Shu-Ying He, and Yong-Jian Lian**

*Guangzhou Maternal and Neonatal Hospital (Y.-L.F., Y.-K.L., S.-Y.H., Y.-J.Li.), Guangzhou Medicinal Institute (Y.-X.H., T.L., B.K., Y.-J.Lu.), Sun Yat Sen University of Medical Sciences (H.-L.L.), and Guangzhou Medical College (Z.-Z.Y., M.-G.Z.), Guangzhou, China*

ABSTRACT

**Objective:** The objective of this study was to determine whether human papillomavirus (HPV) infections are involved in the development of papillomatosis lesions of the lower female genital tract.

**Methods:** A total of 616 biopsy specimens of genital papillomatous lesions (307 nodular and 309 papular types) from 598 patients were anaylyzed for the presence of HPV DNA sequences by polymerase chain reaction (PCR). These specimens were also examined by histopathological assessment for characteristic HPV-associated cytological changes, by immunohistochemical staining for HPV-associated antigen, and by electron microscopy for the presence of virions.

**Results:** HPV DNA sequences were found in 97.9% (140 of 143 cases) and 1.1% (1 of 91 cases) of the nodular and papular papillomatosis cases tested, respectively. In 18 patients who had both types of papillomatosis lesions, HPV DNA was invariably found only in nodular tissues. HPV-associated antigen, koilocytosis, and virions were found in 53.6% (98 of 183 cases), 70.5% (129 of 183 cases), and 5.9% (5 of 85 cases) of nodular papillomatosis lesions tested, respectively.

**Conclusions:** These data suggest that nodular papillomatosis was closely associated with HPV infection, but that papular papillomatosis of the lower female genital tract may have an etiology other than HPV infection. © 1994 Wiley-Liss, Inc.

KEY WORDS

Condyloma acuminatum, pseudocondyloma, human papillomavirus, PCR

Human papillomaviruses (HPVs) are found frequently in the female lower genital tract where they induce different lesions depending on the HPV types involved.[1] The association between the presence of different types of HPV and clinical lesions is better established for the cervix than for the vulva. For example, vulvar vestibular papillomatosis is one type of vulvar lesion whose origin and clinical significance remain controversial. Friedrich[2] attributed the vestibular papillae present in asymptomatic women as anatomic and/or functional variants of normal vulvar epithelium. Pekham et al.[3] and Friedrich[4] considered vestibular papillomatosis a condition reactive to phylogosis caused by non-viral agents. However, recent descriptions of so-called "subclinical" HPV infections by Growdon et al.[5] and by Campion et al.[6] raised the question of whether all these vestibular papillae were indeed related to HPV infections.[7–11]

Vulvar condyloma acuminatum, on the other hand, is clearly caused by infection with certain types of HPV that are mainly transmitted through sexual contact.[12–15] Inquiries into the natural history and establishment of an unequivocal diagnosis

Address correspondence/reprint requests to Dr. Yu-Liang Fu, Associated Chief, Guangzhou Maternal and Neonatal Hospital, 402 Renminzong Road, Guangzhou 510180, China.

Received September 14, 1993
Accepted January 15, 1994

Basic Science Article of HPV infection in vulvar vestibular papillomatosis have been hampered by the lack of an in vitro propagation system for the virus and by the poor correlation between HPV serology and disease activity. In view of the rapid spreading of HPV infection in recent years, both in Western countries[7,12–18] and in China,[19,20] a reliable method for the detection of HPV infection and differential diagnosis between condyloma acuminata and vulvar vestibular papillomatosis would help patient evaluation and management.

In this paper, we report results of clinical and laboratory examinations of papillomatosis lesions of the lower female genital tract. The polymerase chain reaction (PCR) technique, which was used to amplify HPV DNA sequences and to determine the presence of HPV DNA sequences, was supplemented with histopathological assessments of HPV infections by immunohistochemical staining of HPV-associated antigen, microscopy (both light and electron microscopes), colposcopy, clinical evaluation, and follow-up.

**TABLE 1. Clinical and laboratory findings on nodular and papular papillomatosis lesions in the lower female genital tract***

| Test item | Condyloma acuminatum (nodular type lesions) | Pseudocondyloma (papular type lesions) |
|---|---|---|
| HPV DNA (types 6, 11, 16, 18, and 33) | 97.9% (140/143) | 1.1% (1/91) |
| HPV-associated antigen | 53.6% (98/183) | 0.9% (2/225) |
| HPV virions | 5.9% (5/85) | 0 (0/23) |
| History of multiple sexual partners | 80.8% (248/307) | 7.1% (22/309) |

*Results were presented as percentage of cases tested positive. Differences in all test items (except HPV virions) between these two groups of genital papillomatosis were statistically significant ($P < 0.01$).

### SUBJECTS AND METHODS

### Patients and Specimens

Between January 1990 and August 1992, a total of 616 vulvar papillomatosis biopsies (307 nodular and 309 papular types) were obtained from 598 patients, including 18 patients who had both nodular and papular types of vulvar papillomatosis, before any therapeutic management was implemented. The ages of these patients were between 14 and 48 years. Laboratory tests were performed and results were analyzed in a double-blinded fashion.

### Determination of HPV DNA Sequences by PCR

Portions of freshly frozen tissues were treated with proteinase K (Sigma Chemicals, St. Louis, MO). DNA was extracted by phenol and chloroform and purified by alcohol precipitation. Purified DNA was then used as template for PCR amplification of HPV DNA sequences. Consensus primers MY09 and MY11 commercially obtained from Perkin-Elmer Cetus (Norwalk, CT) are capable of amplifying DNA from genital HPV types 6, 11, 16, 18, 31, 33, 35, 39, 40, 42, 45, 51, 52, 53, 54, 55, 57, and 59 and from at least another 25 yet uncharacterized HPV types.[18] Dermal HPV types 1, 5, 8,26, 27, 41, 47, and 48 are also amplified with these primers. Sequences of the oligonucleotide primers were CGTCCMARRGAWACTGATC and GCMCAGGWCATAAYAATGG (where M = A + C, R = A + G, W = A + T, Y = C + T). DNA samples, deoxyribonucleoside triphosphates, and primers were heated in buffer to 95°C for 5 min before *Taq* DNA polymerase (Perkin-Elmer Cetus) was added to the reaction mixture and reaction started in a thermocycler (Model 480, Perkin-Elmer Cetus). The temperatures of the reaction mixture were cycled 35 times through 95°C denaturation (50 sec), 50°C annealing (50 sec), and 72°C extension (1 min) with a 5-min incubation at 72°C at the end. Positive and negative control DNA were always included in every PCR assay. Portions of the amplified reaction mixture were separated by electrophoresis in 2% agarose gel with pGEM-3 DNA digested with a mixture of restriction endonucleases *Hinf*I, *Rsa*I, and *Sin*I used as size standards. The HPV positivity was determined by the presence of 450 base pairs of amplified HPV DNA on visual inspection under ultraviolet light after staining with ethidium bromide.[11] The types of HPV were determined by hybridization of amplified DNA with a radioactively labeled type-specific internal oligonucleotide as probe. The sensitivities of detection for HPV by our PCR method were determined by amplifying either a serial dilution of purified cloned HPV DNA of known concentrations or DNA from CaSki and HeLa cells.

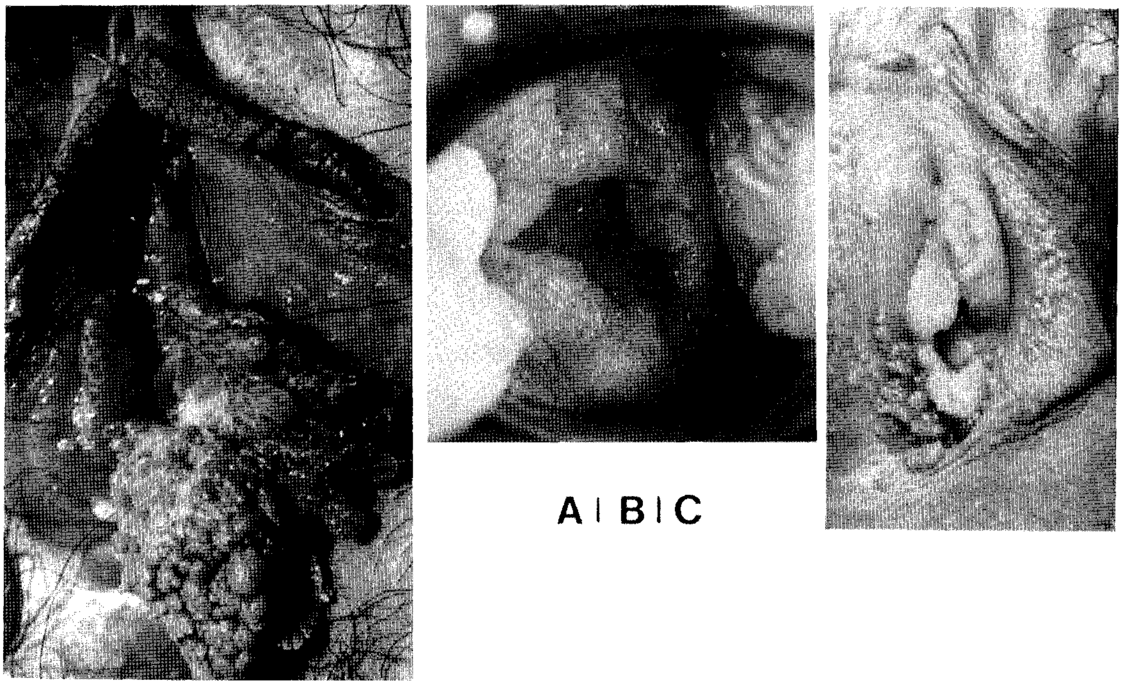

Fig. 1. Gross features of nodular papillomatosis lesions in bunched grapes (A), flat warty (B), and polyps (C) appearances.

### Detection of HPV-Associated Antigen by Avidin Biotin Complex (ABC) Immunochemical Staining Method

ABC reagents were purchased from Vector Laboratories, Inc. (Burlingame, CA) and diluted 1:100 before use. Rabbit antisera directed against L1 gene product of HPV (DAKO B580) and biotin-labeled sheep anti-rabbit IgG antibodies were both diluted 1:200 before use. The presence of intranuclear brownish-yellow granules after development with diaminobenzidine (DAB) was considered an indication of HPV-associated antigen.[21]

### Microscopic Investigations

Light microscopic examinations of koilocytotic changes of HPV-infected cells were performed after routine hematoxylin-eosin (H&E) staining.[12] Koilocytes are intermediate or mature squamous cells characterized by a large perinuclear cavity. Near the cavity, which has sharply defined borders, the cytoplasm is dense and often amphophilic. Nuclei may be single or multiple. The nuclear membrane is not apparent. In the fully developed koilocyte, the chromatin is often smudged. These cells often contain no nucleoli or inclusion bodies. Electron microscopic studies of HPV virions were carried out with ultrathin sections of tissue slices prepared by standard procedures and examined in a JEM (Tokyo, Japan) 100CXII transmission electron microscope after being negatively stained with phosphotungstic acid.[22]

### Clinical Management and Follow-Up

No treatment was given to those asymptomatic patients with papular papillomatosis. All patients were examined every 2 weeks for at least 3 months.

### RESULTS

The clinical and laboratory features and characteristics of nodular and papular types of lesions of the lower female genital tract were quite different. As shown in Table 1, the differences in HPV prevalence, presence of HPV-associated antigen, koilocytotic changes, and history of multiple sexual partners between these two types of papillomatosis were all statistically significant ($P < 0.01$).

Of the 307 women with nodular papillomatosis, 271 (88.3%) had multicentric involvement of the lower genital tract (vagina, cervix, and perineum), 25 (8.1%) had lesions confined to the cervix, and 11 (3.6%) had lesions confined to the vagina. Gross appearances of these nodular papillomatosis cases

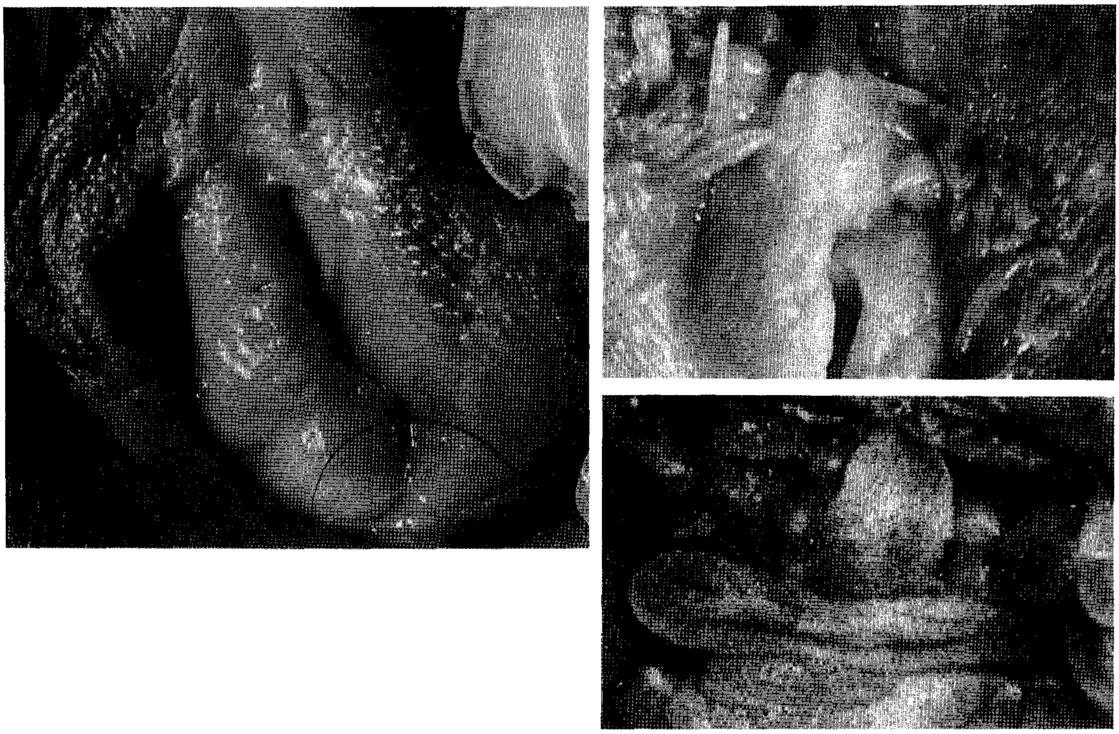

Fig. 2. Clinical features of papular papillomatosis. A: Teardrop pattern; B: finger-like papillae usually 5–8 mm in length; and C: patterned vascular architectures under colposcope.

consisted of those shaped like polyps, chicken-crowns, or bunched grapes found in lower genital tract and perineum areas (272 cases); fuzzy-ball appearances found in the vestibular area (8 cases), and flat warty appearances found in the cervix (20 cases) (Fig. 1). The gross appearances of the 309 cases of papular papillomatosis were mostly soft and smooth teardrops or roughened and raised mucosal surfaces (Fig. 2A) or finger-like (Fig. 2B) projections with patterned vascular architectures on colposcopy (Fig. 2C). Among 309 cases of the papular lesions, 279 (90.3%) were found on the inside mucosal surface of the labia minora and 30 (9.7%) were found in the vagina.

HPV DNA sequences of one or more types of HPV could be detected in 140 of 143 (97.9%) cases of nodular papillomatosis lesions tested by the PCR method (Fig. 3). Repeated testing of the 3 HPV negative cases was not possible because no papillomatosis tissue was left after the first biopsy. On the other hand, only 1 of the 91 (1.1%) papular papillomatosis tissue tested positive for HPV DNA with the PCR method, and this single case became negative when tested again a week later. Four of the HPV DNA negative cases were tested negative 2 or 3 times upon patients' requests within a 6-month period. Noteworthy were the HPV DNA PCR test results on specimens obtained from 18 patients who were found to have both nodular and papular types of papillomatosis on the mucosal surface of their labia minora or lower vagina. HPV DNA sequences were found in all 18 nodular portions but in none of the papular portions of the lesions. Two women with sparse and very minute lesions of uncertain classification who tested positive for HPV DNA sequences had developed nodular lesions when examined again 2 weeks later.

HPV-associated antigen could be stained by the immunohistochemical method in 98 of 183 cases (53.6%) of nodular lesions tested but in only 2 of 225 cases (0.9%) of papular type of papillomatosis lesion examined with the same method (Fig. 4). No characteristic koilocytotic changes were seen in the papular papillomatosis tissues. Other microstructural features in the cells of the nodular papillomatosis included the disappearance of cellular organelles, enlargement of mitochondria, and vacuole formation in the nuclei. Electron microscopy re-

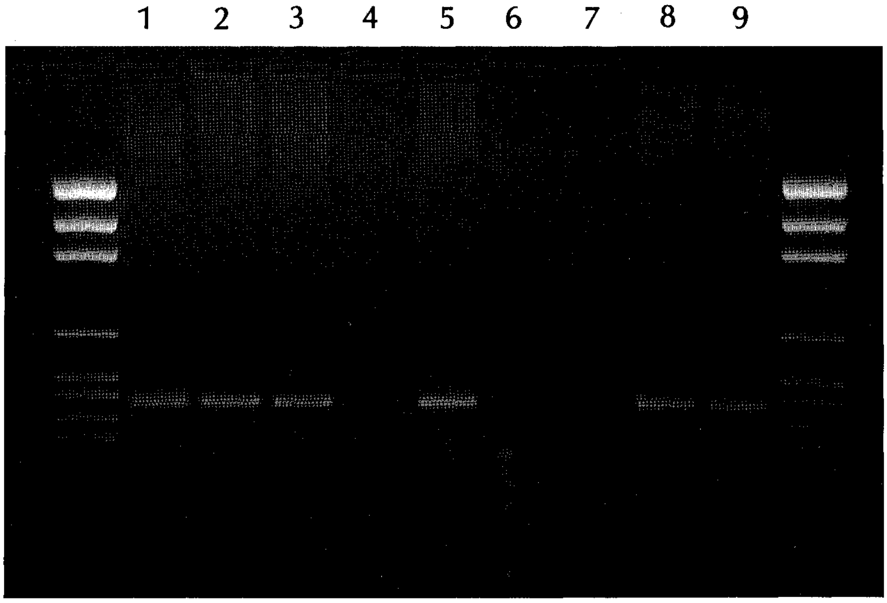

Fig. 3. Agarose gel electrophoresis of amplified HPV DNA using consensus primers and under conditions described in "Subjects and Methods." pGEM-3 DNA digested with a mixture of restriction endonucleases *Hinfl*, *Rsal*, and *Sinl* was used as size standards in the 2 outside lanes, and the sizes are (from top to bottom) 2,645, 1,605, 1,198, 676, 517, 460, 396; 350, 222, 179, 126, 75, 65, 51, and 36 base pairs. **Lanes 1,2:** Amplification of DNA from purified cloned HPV types 6 and 11 DNA, respectively. **Lanes 3,4:** Amplification of nodular and papular papillomatosis tissues from 2 patients, respectively. **Lanes 5,6:** Amplification of DNA from nodular and papular portions of papillomatosis tissues of a single patient, respectively. **Lane 7:** Amplification of 50 ng of human DNA with the HPV consensus primers MY09 and MY11. **Lanes 8,9:** Amplification of CaSki and HeLa cells, respectively, which are known to contain HPV types 16 and 18, respectively.

vealed a grid pattern and round HPV virion-like structure of 40–50 μm in diameter in 5 of 85 cases (5.9%) of nodular papillomatosis lesions examined (Fig. 5). No such virion-like structure could be found in 23 papular papillomatosis tissues.

All 7 cases of papular papillomatosis from patients who were pregnant regressed spontaneously within 1–42 days (mean 14 days) after pregnancies were terminated by abortion or induced or term delivery, but none of the 8 cases of pregnant women with nodular papillomatosis regressed spontaneously during the same period after termination of pregnancy. None of the 89 patients with asymptomatic and untreated papular papillomatosis developed nodular papillomatosis during follow-up of 3 months to 2 years (mean 4 months). No case of genital warts could be documented among sexual partners of patients with papular papillomatosis. A history of multiple sexual partners was admitted by 248 of 307 (80.8%) patients with nodular papillomatosis, but by only 22 of 309 (7.1%) patients with papular papillomatosis. Furthermore, 23 of 309 patients with papular papillomatosis were young virginal women in their 20s with intact hymen upon physical examinations.

### DISCUSSION

Research in the pathogenesis of HPV in the past has concentrated on the carcinogenic nature of HPV and on the development of condyloma acuminatum. Relatively little is known so far on whether HPV infection is really involved in the development of vestibular papillae or papular papillomatosis of the lower female genital tract. The term "pseudocondyloma" based on HPV-associated antigen studies and the term "condyloma-like vulvar lesions" based on pathophysiological studies on koilocytotic cells have been used synonymously to describe these lesions.[10] Various other terminologies have also been found in the literature to describe these papular papillomatosis lesions which include "subclinical HPV infections," "precondyloma," and condyloma.[7–9] There is no consensus on the possible origin and etiology of these lesions,

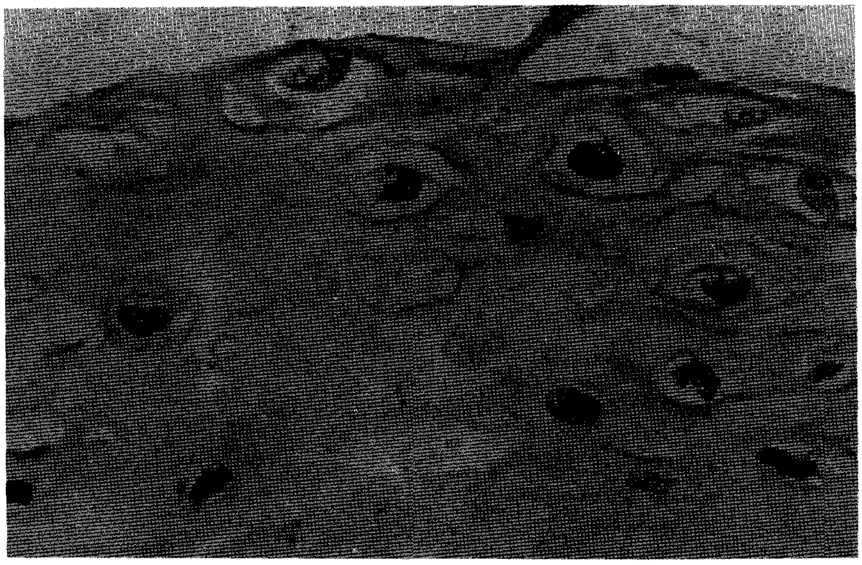

**Fig. 4.** Immunohistochemical staining of HPV L1 gene antigen by ABC method. Presence of HPV L1 gene antigen is indicated by granules inside nuclei that stained brownish-yellow with DAB.

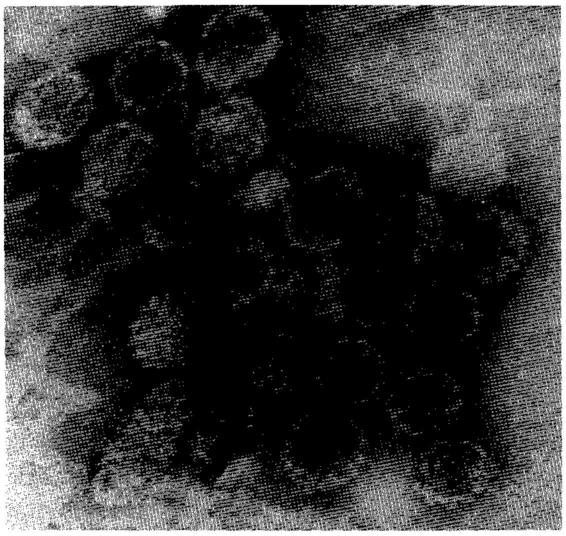

**Fig. 5.** HPV virions under electron microscope. ×330,410.

although it has been reported that patients with vulvar micropapillomatosis are not significantly more frequently infected with HPV than controls.[23] The PCR method used in this study has a lower limit of detection of approximately 50–500 viral genomes equivalent (based on HPV types 6, 11, 16, and 18 results) depending on the type of HPV. Our PCR results indicated that the HPVs present in almost all nodular lesions but were either present in extremely small quantity (estimated to be less than 1 copy per 3,000 cells) or, more likely, not present in papular lesions at all. Because of its technically demanding and labor-intensive nature, electron microscopy has probably only very limited use in the diagnosis of HPV infections.

In summary, HPV DNA sequences, HPV-associated antigen, koilocytotic changes, and virions can be found very frequently in nodular papillomatosis lesions of the lower female genital tract. These lesions can be transmitted efficiently by sexual contact. On the other hand, our results also clearly suggest that papular papillomatosis lesions very rarely contain HPV DNA sequences. We believe that these HPV DNA results, when taken together with other laboratory tests carried out in this study, suggest that prudence should be exercised in interpreting the nature and origin of papular papillomatosis lesions, especially in asymptomatic women without other clinical indications.

### ACKNOWLEDGMENTS

The authors gratefully acknowledge the encouragement and support of Dr. MengHao Fan of the Public Health Bureau of Guangzhou.

### REFERENCES

1. Howley P: Role of the human papillomaviruses in human cancer. Cancer Res (Suppl) 51:5019s–5022s, 1991.
2. Friedrich EG: The vulvar vestibule. J Reprod Med 28: 773–777, 1983.
3. Pekham BM, Maki DG, Patterson JJ, Hafez GR: Focal vulvitis: A characteristic syndrome and cause of dyspareunia. Am J Obstet Gynecol 154:855–864, 1986.
4. Freidrich EG: Vulvar vestibulitis syndrome. J Reprod Med 82:110–114, 1987.
5. Growdon WA, Fu YS, Lebherz TB, Rapkin A, Mason GD, Parks G: Pruritic vulvar squamous papillomatosis: Evidence for human papillomavirus etiology. Obstet Gynecol 66:564–568, 1985.
6. Campion MJ, Cuzick J, McCance DJ, Singer A: Progressive potential of mild cervical atypia: Prospective cytological, colposcopic and virological study. Lancet 2:237, 1986.
7. Sobel JD: Vulvovaginal infections: Current concepts in diagnosis and therapy. In Reid R (ed): The Biology and Management of Human Papillomavirus Associated Diseases. New York: Academy Professional Information Services, pp 108–148, 1990.
8. Cecchini S, Grazzini G, Iossa A, et al: Subclinical vulvar papillomavirus infection. J Reprod Med 36:143–146, 1991.
9. Costa S, Rotala A, Terzano P, et al.: Is vestibular papillomatosis associated with human papillomavirus? J Med Virol 35:7–13, 1991.
10. Nuovo GJ, O'Connell M, Blanco JS, Levine RU, Silverstein SJ: Correlation of histology and human papillomavirus DNA detection in condyloma acuminatum and condyloma-like vulvar lesions. Am J Surg Pathol 13:700–706, 1989.
11. Cone R, Beckmann A, Aho M, et al.: Subclinical manifestations of vulvar human papillomavirus infection. Int J Gynaecol Pathol 10:26–35, 1991.
12. Sehgal VN, Koranne RV, Srivastava SB: Genital warts. Current status. Int J Dermatol 28:75–85, 1989.
13. Beutner KR, Becker TM, Stone KM: Epidemiology of human papillomavirus infections. Dermatol Clin 9:211–218, 1991.
14. Howley PM, Schlegel R: The human papillomavirus. An overview. Am J Med 85:155–158, 1988.
15. World Health Organization: Genital human papillomavirus infection and cancer. Memorandum from a WHO meeting. Bull WHO 65:817–827, 1987.
16. Patsner B, Baker DA, Orr JW Jr: Human papillomavirus genital tract infections during pregnancy. Clin Obstet Gynecol 33:258–267, 1990.
17. Hatch KD: Vulvovaginal human papillomavirus infections: Clinical implications and management. Am J Obstet Gynecol 165:1183–1188, 1991.
18. Bauer HM, Ting Y, Greer CE, et al.: Genital human papillomavirus infections in female university students as determined by a PCR-based method. JAMA 265:472–477, 1991.
19. National Sexually Transmitted Disease Surveillance Working Team: Brief report and analysis on the prevalence of sexually transmitted diseases from national surveillance stations between 1987 and 1990. Beijing, China: Ministry of Health, China 54:2–5, 1991.
20. Fu YL, Chun JZ, Chiu TM, Sun CK: Clinical analysis of 5,905 cases of sexually transmitted diseases in Guangzhou. Chin J Obstet Gynecol 25:262–265, 1990.
21. Wilbur DC, Reichman RC, Stoler MH: Detection of infection by human papillomavirus in genital condyloma. A comparison study using immunochemistry and in situ nucleic acid hybridization. Am J Clin Pathol 89:505–510, 1988.
22. Lee FK, Nahmias AJ, Stagno S: Rapid diagnosis of cytomegalovirus infection in infants by electron microscopy. N Engl J Med 299:1266–1270, 1978.
23. Bergeron C, Ferenczy A, Richart R, Guralinick M: Micropapillomatosis labialis appears unrelated to human papillomavirus. Obstet Gynecol 76:281–286, 1990.
